# *Ex Vivo* and *In Vivo* Imaging and Biodistribution of Aptamers Targeting the Human Matrix MetalloProtease-9 in Melanomas

**DOI:** 10.1371/journal.pone.0149387

**Published:** 2016-02-22

**Authors:** David Kryza, Frédéric Debordeaux, Laurent Azéma, Aref Hassan, Olivier Paurelle, Jürgen Schulz, Catherine Savona-Baron, Elsa Charignon, Pauline Bonazza, Jacqueline Taleb, Philippe Fernandez, Marc Janier, Jean Jacques Toulmé

**Affiliations:** 1 UNIV Lyon, Université Claude Bernard Lyon 1, LAGEP UMR 5007 CNRS, Villeurbanne, France; 2 Hospices Civils de Lyon, Imthernat plateform, Lyon, France; 3 Université de Bordeaux, CNRS, INCIA, UMR 5287, Bordeaux, France; 4 Service de Médecine Nucléaire, CHU de Bordeaux, Bordeaux, France; 5 Inserm U1212, Bordeaux, France; 6 CNRS UMR5320, Pessac, France; 7 University of Bordeaux, ARNA Laboratory, Bordeaux, France; IDI, Istituto Dermopatico dell'Immacolata, ITALY

## Abstract

The human Matrix MetalloProtease-9 (hMMP-9) is overexpressed in tumors where it promotes the release of cancer cells thus contributing to tumor metastasis. We raised aptamers against hMMP-9, which constitutes a validated marker of malignant tumors, in order to design probes for imaging tumors in human beings. A chemically modified RNA aptamer (F3B), fully resistant to nucleases was previously described. This compound was subsequently used for the preparation of F3B-Cy5, F3B-S-acetylmercaptoacetyltriglycine (MAG) and F3B-DOTA. The binding properties of these derivatives were determined by surface plasmon resonance and electrophoretic mobility shift assay. Optical fluorescence imaging confirmed the binding to hMMP-9 in A375 melanoma bearing mice. Quantitative biodistribution studies were performed at 30 min, 1h and 2 h post injection of ^99m^Tc-MAG-aptamer and ^111^In-DOTA-F3B. ^99m^Tc radiolabeled aptamer specifically detected hMMP-9 in A375 melanoma tumors but accumulation in digestive tract was very high. Following i.v. injection of ^111^In-DOTA-F3B, high level of radioactivity was observed in kidneys and bladder but digestive tract uptake was very limited. Tumor uptake was significantly (student *t* test, p<0.05) higher for ^111^In-DOTA-F3B with 2.0%ID/g than for the ^111^In-DOTA-control oligonucleotide (0.7%ID/g) with tumor to muscle ratio of 4.0. Such difference in tumor accumulation has been confirmed by *ex vivo* scintigraphic images performed at 1h post injection and by autoradiography, which revealed the overexpression of hMMP-9 in sections of human melanomas. These results demonstrate that F3B aptamer is of interest for detecting hMMP-9 in melanoma tumor.

## Introduction

Among oncological physiopathologic processes, angiogenesis appears to be a promising way for targeted therapies [1]. In this process, matrix metalloproteinases (MMP) are one of main actors of degradation of the extracellular matrix and basement membrane, facilitating tumor cell invasion [2]. Among them, MMP-9 is over-expressed in numerous malignant tumors [3] and particularly in cutaneous malignant melanoma [4–5].

Malignant melanoma is a tumor that arises from melanocytic cells and primarily involves the skin but it can also arise in the eye, meninges and on various mucosal surfaces. It can metastasize either by the lymphatic or haematogenous way. Distant metastases have a poor prognosis with no long-term curative treatment [6] and with a median survival in untreated patients being only 6–9 months. Efficient and accurate diagnosis is highly needed for this pathology. In high-risk patients, computerized tomography with or without FDG-positron emission tomography (FDG-PET) and magnetic resonance imaging may be indicated depending on the clinical findings. Nevertheless, those techniques lack specificity with relatively high false-positive rate and a low sensitivity for the detection of occult regional nodal metastases. Molecular imaging techniques, using specific target marker, are needed for *in vivo* mapping and measuring pathological processes at cellular or even molecular levels.

Developped in the early 1990s [7–9], aptamers are three-dimensional oligonucleotides, either with a deoxy- (DNA) or a ribo-scaffold (RNA). They bind with high affinity and high specificity to a large variety of targets ranging from small organic molecules to viruses and live cells [10–12], by interaction optimized by shape complementarity. Aptamers are generated by an iterative process of *in vitro* selection and amplification called SELEX (Systematic Evolution of Ligands by EXponential enrichment) [13] and can be chemically-modified thus generating powerful tools for biomedical applications [14]. Particular interest is being paid to aptamers obtained by cell-SELEX, recognizing membrane proteins and receptors [15–17]. A number of reports describe aptamers specific of tumor markers [18–19]. Such aptamers are used for many different purposes such as biosensing [20], delivery [21–22], flow-cytometry [23], diagnostics [24–25] and therapeutics [21, 26]. The binding characteristics of aptamers, namely high affinity and specific recognition of the target as well as their easy of synthesis made them excellent candidates for imaging. Aptamer-based probes have been engineered for monitoring several tumor markers: nucleolin [27], tenascin [28], PSMA [29], PTK7 or MUC1 glycoprotein [30–31], annexin A2 [32] or MMP-9 [33]. The versatility of oligonucleotide synthesis allows the chemical modifications od aptamers at defined positions without effect on their binding properties. Indeed different imaging modalities were implemented with aptamer probes: Magnetic Resonance Imaging (MRI) [34], optical imaging [30; 35] or nuclear imaging probe [36–37]. Promising results are available notably for the *in vivo* detection in animal models of tenascin [28], nucleolin [27] or MUC1 glycoprotein [31].

The F3B aptamer that we previously raised against the human MMP-9 exhibits the desired properties for an imaging agent: affinity in the low nanomolar range, high specificity of binding and full nuclease resistance due to a chemically modified backbone [33]. We previously demonstrated its interest for *ex vivo* imaging sections of human brain without any further *in vivo* evaluation. In the present study, we evaluated the *ex vivo* and *in vivo* melanoma tumor targeting efficiency of F3B towards hMMP-9 protein using a fluorescent or isotope labelled aptamer.

## Materials and Methods

### Oligonucleotide Synthesis

F3B and the control sequence, bearing a 5′ hexylamino function, were synthesized on a 1 μmol scale with an ABI Expedite 8909 synthesizer, using conventional β-cyanoethyl phosphoramidite chemistry (2’OMe-purine and 2’F-pyrimidine). Once purified (electrophoresis on denaturating gels: 20% (19:1 acrylamide/bis-acrylamide), 7M urea, Tris-Borate-EDTA buffer), oligonucleotides were conjugated to DOTA or Cy5, according to a previously described protocol for MAG_3_ coupling [33]. Briefly, 20 nmol of oligonucleotide were suspended in 100 μL of binding buffer (sodium bicarbonate/sodium carbonate 0.25 M, pH 8.3, sodium chloride 1 M, sodium ethylenediaminetetraacetate 1 mM) and gently stirred at room temperature. DOTA-NHS (Chematech^®^) or Cy5-NHS (Interchim^®^) (3 mg, in 30 μL of DMF) was added in portions at room temperature over 3 h. After complete addition, the suspension was stirred for an additional hour, and the crude was directly purified by HPLC (Macherey-Nagel Nucleodur column, 0.1 M triethylammonium acetate, pH 7.0, (acetonitrile/0.1 M triethylammonium acetate, pH 7.0: 80/20) gradient) to afford the oligonucleotide conjugates in 50−90% yield. Conjugate characterization was performed through MALDI-ToF mass spectrometry (Reflex III, Bruker) or Electrospray ionization (LCT Premier, Waters).

### Protein

The human MMP-9 was purchased from Calbiochem; samples were checked for purity by SDS polyacrylamide gel electrophoresis.

### Specificity Assays

#### Surface Plasmon Resonance

The specificity of F3B-DOTA and F3B-Cy5 derivatives for hMMP-9 was determined through competition with the F3B unmodified aptamer monitored by surface plasmon resonance (BiaCore 3000 apparatus, BiaCore AB, Sweden). 5’-biotin F3B and its control were immobilized on a Xantec^™^ SAD 200m chip (50 μL of 50 nM solution in PBS buffer, at a rate of 20 μL/min). HMMP9 was firstly injected at 20 μL/mn on F3B and control channel. Then hMMP-9 (50 nM in PBS buffer) was incubated with F3B or with the control labelled derivatives (200 nM in PBS buffer) and injected on F3B-immobilized channel (80 μL, 20 μL/min) at 23°C. The complexes were dissociated with a pulse of a solution containing 40% formamide/3.6 M urea/30 mM EDTA.

#### Electrophoretic Mobility Shift Assay

MMP9/F3B-Cy5 complex was visualized on acrylamide/bis-acrylamide 37.5/1 8% native gel. The polymerization and electrophoresis buffer was TBE 0.5X (Euromed). F3B-Cy5 (20nM) and MMP9 (100nM) were allowed to form the complex at room temperature. Increasing concentration of competitor (0-100nM) was then added and the samples were loaded onto the gel after 30 minutes of incubation. Electrophoresis was performed on a cooled support (4°C) at 150 V. The fluorescence measurement was performed on a Imagequant (LAS4000, GE) at 635 nm for excitation and 670 nm for emission. Intensity of the shift band was calculated with ImageJ software.

### Oligonucleotide sequences

2’OMe-R; 2’F-Y; X = hexylamino phosphate; Y = hexaethyleneglycol phosphate

F3Bomf-NH_2_. 5’-XY UGC CCU GCC CUC ACC CGU UAG CCU GAG CGC CCC GCA-3’

Control-NH_2_. 5’-XY UGC CAA ACG CGU CCC CUU UGC CCG GCC UCC GCC GCA-3’

### Oligonucleotides Radiolabelling

#### Technetium 99m labelling

F3B-MAG3-aptamer and MAG3-control-sequence were labelled by modifying an existing protocol. Briefly, the oligonucleotides (20 μg) were dissolved in phosphate buffer saline (PBS, 20 μL) in a sterile vial. Then 20 μL of a mixture of sodium tartrate (50 mg/mL) in sterile 0.5 M sodium bicarbonate, 0.18 M ammonium hydroxide, 0.25 M ammonium acetate were added to the vial following by immediately adding 2 μL of a freshly prepared SnCl_2_.2H_2_O solution in HCl 0.1 N (1 mg.mL^-1^). ^99m^TcO_4_^-^ was eluted as a physiological saline solution from a commercial ^99^Mo/^99m^Tc generator obtained from Cisbio-international (Saclay, France). 74 MBq of ^99m^TcO_4_^-^ was then added to the mixture, which was incubated 20 min at room temperature. The radiolabeling mixture was then purified from radiochemical impurities by steric exclusion chromatography through a PD-10 column (GE Healthcare Bio-Sciences AB, Uppsala, Sweden) as follows. The column was first washed with 15 mL of isotonic saline and the mixture solution was then loaded on the column followed by elution with isotonic saline. The radiochemical purity (RCP) was assayed with a gamma isotope TLC analyzer (Raytest, Courbevoie, France) using ITLC-SG (Biodex Tec-control black, Biodex, NY, USA) and methyletylcetone (MEK) as mobile phase. Finally, the fractions with the highest radiochemical purities were pooled.

#### Indium 111 labelling

F3B-DOTA-aptamer and DOTA-control-sequence (20 μg) were labelled by adding 300 μL of acetate buffer 0.1 M pH5 and 30–70 MBq of high purity ^111^In-chloride at room temperature for 45 min. Free indium 111 was removed using a PD-10 column. The column was first washed with 15 mL of acetate buffer 0.1 M, then the labelled mixture was loaded on the column and eluted using acetate buffer. ^111^In-DOTA-F3B or ^111^In-DOTA-control were first eluted. RCP of each 0.5 mL fraction was evaluated using ITLC-SG (biodex, Tec-control black) and citrate buffer 50 mM (pH 5) as mobile phase. ^111^In-DOTA-F3B or control remained at the origin whereas unbound indium 111 migrated with an Rf of 0.9–1. The highest radiochemical purity fractions were pooled.

#### Stability testing

For stability testing, an aliquot of the purified ^99m^Tc-MAG3-F3B, ^111^In-DOTA-F3B and labelled control-aptamer were incubated at 37°C in 2 mL phosphate buffer saline (pH 7.4) and radiochemical purity was evaluated as described above.

### Biodistribution studies

Biodistribution studies were performed in compliance with the French guidelines and Ethics approval was obtained from the local animal committee of University Claude Bernard Lyon 1. Mice were obtained from Janvier labs (Saint-Berthevin, France). All animals were housed under standard environmental conditions at ambient temperature of 25°C and cared with free access to water and food. For all experiments, mice were anaesthetized using a gaseous protocol (isoflurane / oxygen (2.5%/2.5%). Melanoma bearing mice were obtained by subcutaneous injection of 3.10^5^ cells of human malignant melanoma A375 in the right flank or in the paw of nude mice (6–8 weeks old, 20-25g).

#### Quantitative biodistribution studies

1 to 10 MBq of radiolabeled aptamer in a maximum volume of 100 μL were intravenously injected into mice bearing human melanoma tumors (n = 3 or 4 for each groups). Mice were sacrificed at defined time points: 30 min, 2 and 4 h after injection by cervical dislocation. Tissues of interest (blood, heart, lungs, spleen, kidneys, muscles, brain, and skin) were removed, weighted and radioactivity was counted for 5 min in a gamma scintillation counter (Wizard^®^ gamma counter, Perkin Elmer, USA). Urine and feces were collected thanks to individual metabolic cage for housing animals and counts. Tissue distribution was expressed as the percentage of injected dose per gram (%ID/g). Renal and hepatobiliary elimination were expressed as cumulated radioactivity under total injected activity. Tumor to muscle ratio (TMR) was calculated using the following formula: (%ID/g tumor) / (%ID/g muscle).

#### Blood half-life of radiolabeled aptamer

After i.v. injection of 1–2 MBq of radiolabeled aptamer, blood samples were removed at different time intervals: 2 min, 5 min, 10 min, 15 min, 30 min and 1 hour and were weighted and counted for 5 min in a gamma counter. Data points were fitted to an exponential decay function.

### Imaging protocol

*Ex vivo* planar scintigraphic imaging was performed on posterior paw being removed and positioned over the collimator of a dedicated small animal Nano-SPECT-CT system (BIOSCAN^™^, Washington DC, USA) at 1 hour after i.v. injection of radiolabeled ^111^In-aptamer. The acquisition was performed during 10 minutes with two 15% windows centered on the two peaks 171 keV and 245 keV of indium 111.

#### *In vivo* and *ex vivo* fluorescence imaging

Optical imaging was performed thanks to a back-thinned CCD-cooled camera (ORCAIIBT-512G, Hamamatsu phonics, Massy, France) using a colored glass long-pass RG 665 filter (Melles Griot, Voisins les Bretonneaux, France), which cuts off all excitation light. Dorsal images were acquired 1h after i.v. injection of 100 μL of Cy5-F3B-aptamer or Cy5-control-sequence (1 to 5 nmol) into mice bearing human melanoma tumors. Mice were sacrificed at 1h post injection and tissues of interest (kidneys, liver, brain, spleen, heart, lungs bone, skin muscle, digestive tract and tumor) were imaged.

#### Immunohistochemical analysis

Patients samples used in these studies were obtained after written informed consent in accordance with the Declaration of Helsinki and stored at the “CRB Cancer des Hôpitaux de Toulouse” collection. According to the French law, CRB Cancer collection has been declared to the Ministry of Higher Education and Research (DC-2008-463) and a transfer agreement (AC-2008-820) was obtained after approbation by ethical committees (“Cancéropole Grand Sud Ouest”). Clinical and biological annotations of the samples have been declared to the CNIL (Comité National Informatique et Libertés).

Different types of well-characterized tumors were formalin-fixed and paraffin-embedded (Single Spread Melanoma, Nodular Melanoma, Acral lentiginous melanoma, Lentigo Malignant Melanoma (Dubreuilh’s melanoma), nodes/metastases). Immunohistochemical hMMP-9 detection was performed on serial sections in order to validate radiolabeling analysis, using a purified anti-hMMP-9 mouse monoclonal antibody (ab58803, Abcam). Appropriate positive and negative controls omitting the primary antibody have been included with every slide run.

Representative paraffin embedded 5 μm thick sections were dewaxed with xylene, rehydrated through a graded alcohol series, and washed with distilled water. A blocking step is needed in order to block endogenous peroxidase activity. Moreover, antigen-unmasking procedure was applied to achieve optimum staining.

After washing with TBS, the slides were saturated, and then incubated with the hMMP-9 antibody (ab58803, 1:300) overnight in humidified atmosphere at room temperature. Sections were washed twice with TBS tween 1%, and ImmPRESS peroxydase Reagent kit (rabbit anti-mouse antibody, ImmPRESS^™^, Vector^®^) was then applied as the secondary antibody in moist chamber for 30 min. Tissue sections were stained with AEC (3-Amino-9-EthylCarbazole, ab 64252, Abcam, Cambridge) for 15 min and counterstained with hematoxylin for examination. Immunoreactivity was evaluated in the cell cytoplasm, cytoplasmic membrane, and in the extracellular matrix.

#### Radiolabeling of tumor tissue sections

As for immunohistochemical analysis, binding studies were performed using tumor sections, but incubated in the presence of either ^111^In-DOTA-F3B aptamer or ^111^In-DOTA-control sequence. 5 μm thick slices were deparaffinised and prepared as previously described.

These slices were incubated with ^111^In-DOTA-F3B aptamer and adjacent section with ^111^In-DOTA-control-sequence for 60 min at room temperature (15 kBq/40 μL). Unfixed radioligand was removed with PBS-Tween 0.05% and slices were washed in distilled water. Radioactivity was finally evaluated using a micro-imager 2000 (Biospace Mesures, Paris, France).

## Results

### Characteristics of hMMP-9 aptamer and specifity assays

**Fig 1:**
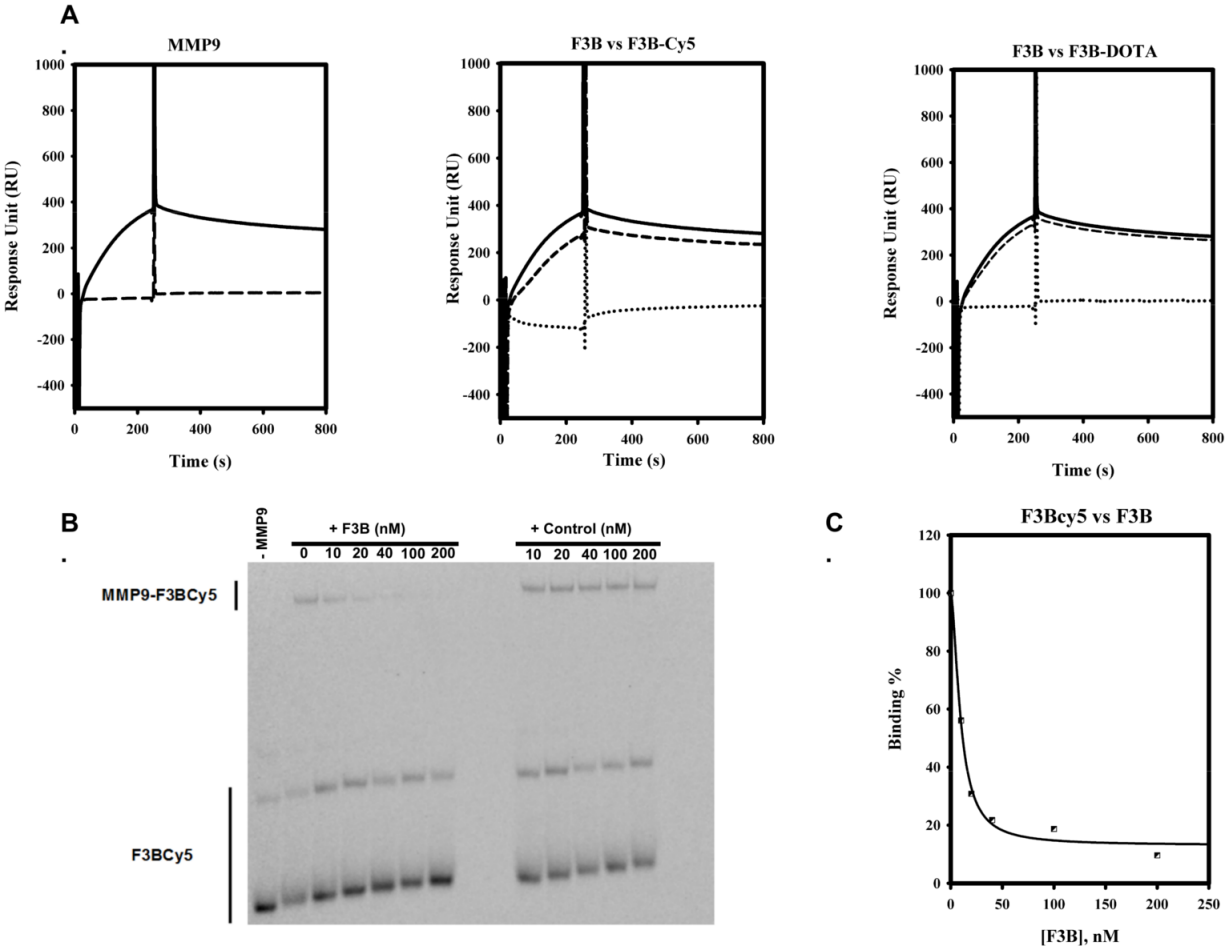
A. Surface Plasmon Resonance assay. Left Panel. MMP-9 (50 nM) was injected at 20 μL/mn on F3B-immobilized channel (solid line) or Control-immobilized channel (dashed lines). MMP-9 forms a complex only in presence of immobilized F3B. Central and right panels: MMP-9 (50 nM) was injected on F3B-immobilized channel at 20 μL/mn alone (solid lines) or after pre-incubation with control conjugates (200 nM, dashed lines) or F3B conjugates (200 nM, dot lines). Pre-incubation with control conjugates (central panel: Cy5, right panel: DOTA) did not abolish complex formation. Whereas pre-incubation with F3B conjugates (central panel: Cy5, right panel: DOTA) led to complex abolition. B. Electrophoretic Mobility Shift Assay. The preformed F3B-Cy5-MMP9 complex was allowed to compete with increasing concentration of competitor (F3B or control). The incubation with F3B competitor led to disappearance of F3B-Cy5-MMP9 shift band whereas incubation with the control did not abolish the F3BCy5-MMP9 complex formation. C. Electrophoretic Mobility Shift Assay. Binding curve of F3B for MMP9, as an integration of F3B-Cy5-MMP9 band intensity. A K_D_ of 15 μM was gathered from the curve.

The solid phase synthesis of oligonucleotides allows the controlled incorporation of appending group anywhere into the aptamer sequence through phosphoramidite chemistry. We opted for the 5’ modification of aptamer, with the display of a reactive amino group, used for conjugation with N-hydroxysuccinimide ester activated labels. This amine function is away from the oligonucleotide part through the incorporation during synthesis of various spacers: a dimer of 2’deoxy-thymidine or a hexaethyleneglycol group. The first spacer was used in the case of cyanine-5 incorporation, whereas the second spacer was used for DOTA ligation. Beside biodistribution modulation problems, we had to firstly confirm that 5’appending groups are not disrupting the F3B-MMP-9 complex formation efficiency. For this purpose, we settled a competition assay, monitored by surface plasmon resonance and electrophoretic mobility shift assay. Results are reported on Fig 1. A biotinylated F3B variant was loaded on the streptavidin sensor chip. This variant was able to detect its target at a usual K_D_ of 10 nM. The pre-incubation of the protein (50 nM) with F3B labelled either with cyanine-5 or DOTA group (200 nM) led to the abolition of complex formation on sensor chip. This result was not observed when the control oligonucleotide was used for pre-incubation. It confirms that variants, F3B-Cy5 and F3B-DOTA, preserve three-dimensional features of the aptamer and that the appending groups doesn’t interfere with the recognition region. As a second control, an electrophoretic mobility shift assay was performed. It clearly demonstrates the specificity of the aptamer, as F3B was able to displace the F3B-Cy5-MMP9 complex whereas the competition with the control did not abolish the complex formation.

### Radiolabelling and stability

After steric exclusion chromatography purification, radiolabeled aptamer were obtained with radiochemical purity (RCP) exceeding 98% and 96% for technetium 99m and indium 111 respectively. Radiochemical yields were 70% for ^99m^Tc-MAG-aptamer and 15–20% for ^111^In-DOTA-aptamer. For both radionuclides at 4 hours after incubation at 37°C in phosphate buffer saline pH 7.4, RCP purity was still greater than 95% indicating a suitable kinetic stability to perform *in vitro* and *in vivo* experiments.

### Biodistribution of ^99m^Tc- MAG-F3B and ^99m^Tc-MAG-control

**Fig 2:**
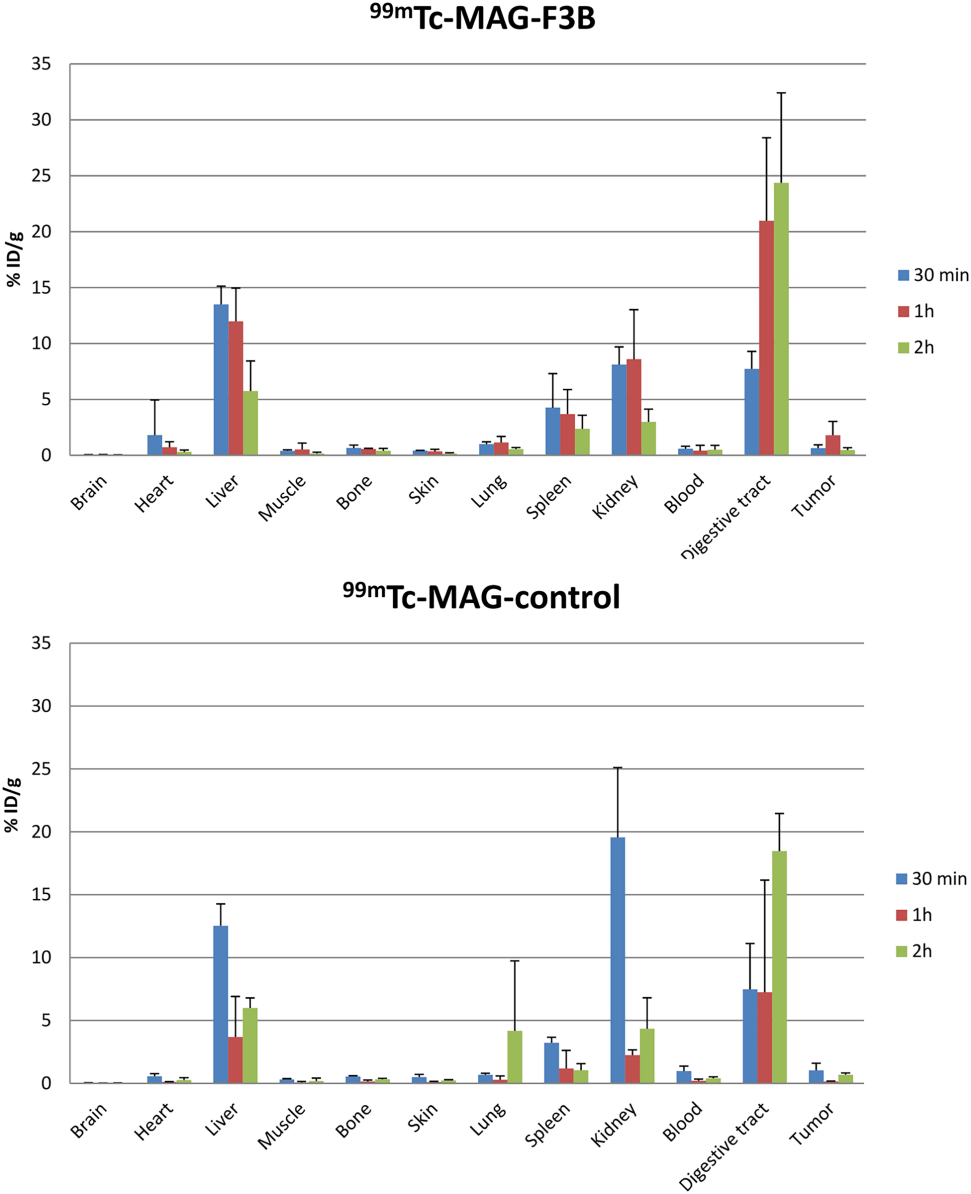
Quantitative biodistribution of ^99m^Tc-MAG-aptamer and ^99m^Tc-MAG-control aptamer as function of post i.v. injection delay expressed as % of injected dose per gram of tissue.

Biodistribution studies were performed in A375 melanoma bearing mice at 30 min, 1 h and 2 h post injection of radiolabeled oligonucleotides and are presented in Fig 2. After i.v. injection of ^99m^Tc-aptamer, high level of radioactivity were observed in kidneys and bladder with more than 48.0 ± 8.8% ID and 60.6 ± 7.7% ID eliminated through renal excretion for ^99m^Tc-MAG-F3B and ^99m^Tc-MAG-control, respectively. High levels of radioactivity were also observed in digestive tract and liver with value of 7.7 ± 1.6%ID/g and 13.5 ± 1.6%ID/g at 30 min post injection and 24.4 ± 8.0%ID/g and 5.7 ± 2.7%ID/g at 2h post injection for ^99m^Tc-MAG-F3B compared to 7.5 ± 3.6%ID/g and 12.5 ± 1.7%ID/g at 30 min post injection and 18.5 ± 3.0%ID/g and 6.0 ± 0.8%ID/g at 2 h post injection for ^99m^Tc-MAG-control indicating also an hepatobiliary clearance of the ^99m^Tc-aptamers. No significant accumulation in other organs was observed for both ^99m^Tc-oligonucleotides at any time. The highest tumor uptake was observed at 1h post injection for the aptamer derivatives with values of 1.8 ± 1.2%ID/g which was significantly higher (Student *t* test, P < 0.05) than for the control ones (0.15 ± 0.03%ID/g). The tumor to muscle ratio (TMR) value for ^99m^Tc-MAG-F3B was 7.6 at 1h post injection respectively compared to 4.4 for the ^99m^Tc-MAG-control.

### Biodistribution of ^111^In-DOTA-F3B and ^111^In-DOTA-control

**Fig 3:**
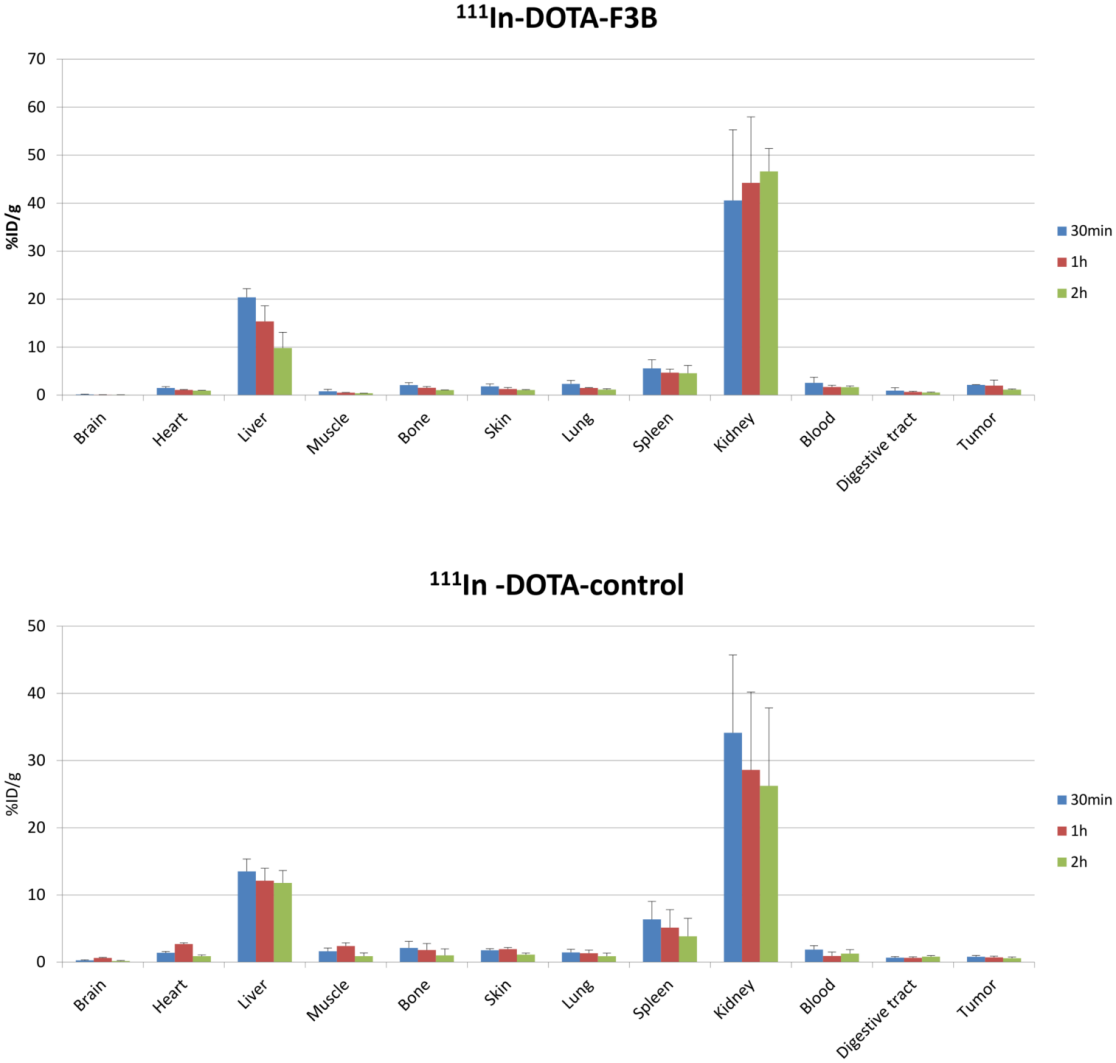
Quantitative biodistribution of ^111^In-DOTA-F3B-aptamer and ^111^In-DOTA-control-aptamer as function of post i.v. injection delay expressed as % of injected dose per gram of tissue.

In order to evaluate the influence of the chelator on the aptamer biodistribution and the tumor targeting, biodistribution studies were performed at 30 min, 1 h and 2 h post injection of ^111^In-DOTA-F3B and ^111^In-DOTA-control. The results are presented in Fig 3. Both aptamers were rapidly cleared from the blood pool (average blood half-life: 11 min) and demonstrated low uptake in normal organs and tissues except from kidneys and liver. The uptake of radiolabeled DOTA aptamers in the digest tract was very low, with value of 0.9 ± 0.6%ID/g and 0.7 ± 0.1%ID/g at 30 min and 1 h post injection, respectively for ^111^In-DOTA-F3B and 0.6 ± 0.2%ID/g and 0.6 ± 0.2%ID/g for ^111^In-DOTA-control at 30 min and 1 h post injection, respectively. Tumor uptake was significantly (student *t* test, p<0.05) higher for ^111^In-DOTA-F3B with values of 2.0 ± 1.1% ID/g at 1 h post injection than for the ^111^In-DOTA-control (0.7 ± 0.2%ID/g). TMR were 4.0 at 1 h post injection for ^111^In-DOTA-F3B and 0.3 for ^111^In-DOTA-control, respectively.

### Imaging studies

**Fig 4:**
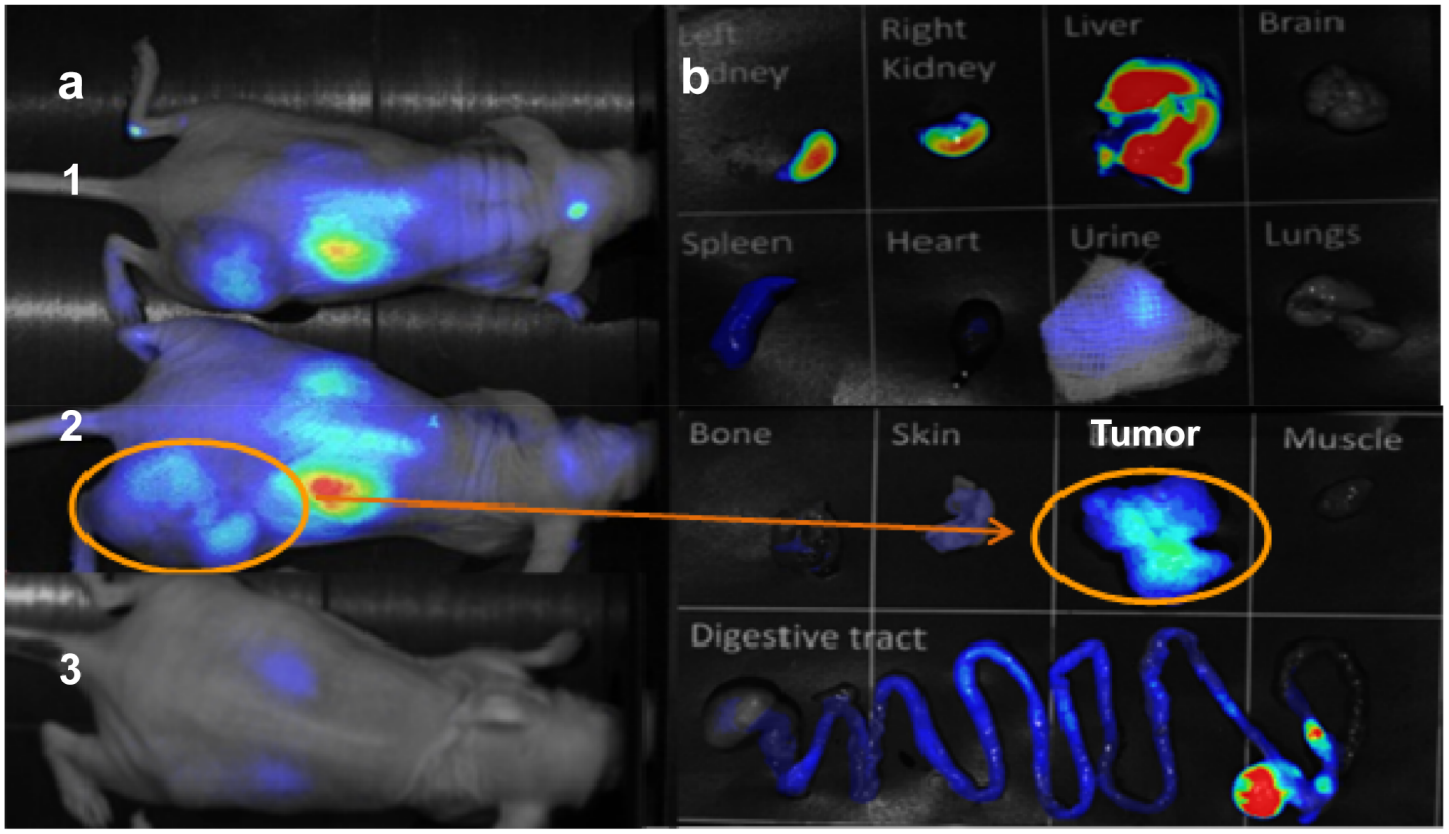
(A) Ventral planar fluorescence reflectance images acquired at 1h post injection of Cy5-F3B-aptamer (1, 2) or Cy5-control-sequence (3) into mice bearing human melanoma tumors. (B) Fluorescence reflectance imaging of organs after dissection of mice 2.

**Fig 5:**
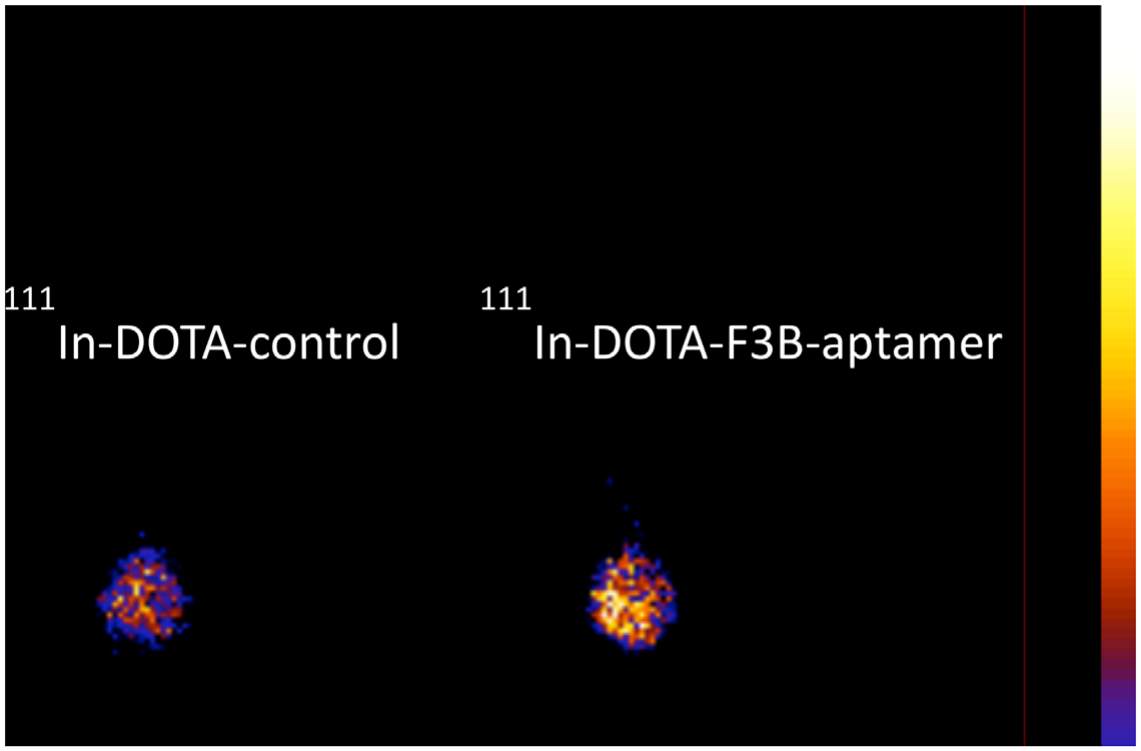
Ex vivo scintigraphic planar image of posterior paw at 1h after i.v. injection of ^111^In-DOTA-F3B-aptamer and 111In-DOTA-control-aptamer.

*In vivo* fluorescent images acquired 1 h after i.v. injection of Cy5-F3B or Cy5-control-sequence into mice bearing human melanoma tumors are presented in Fig 4. Fluorescence staining allowed visualizing the kidneys and the liver and identifying the tumor with Cy5-F3B. Fluorescence imaging of the dissected organs and tumor tissues acquired 1 h after i.v. injection of Cy5-F3B-aptamer revealed well the highest fluorescent organs (i.e.: liver and kidneys) and revealed a moderate signal in the tumor. Such difference in tumor accumulation between F3B and its control has been confirmed by *ex vivo* scintigraphic images performed at 1 h post injection (Fig 5).

### Immunohistochemistry and autoradiography

**Fig 6:**
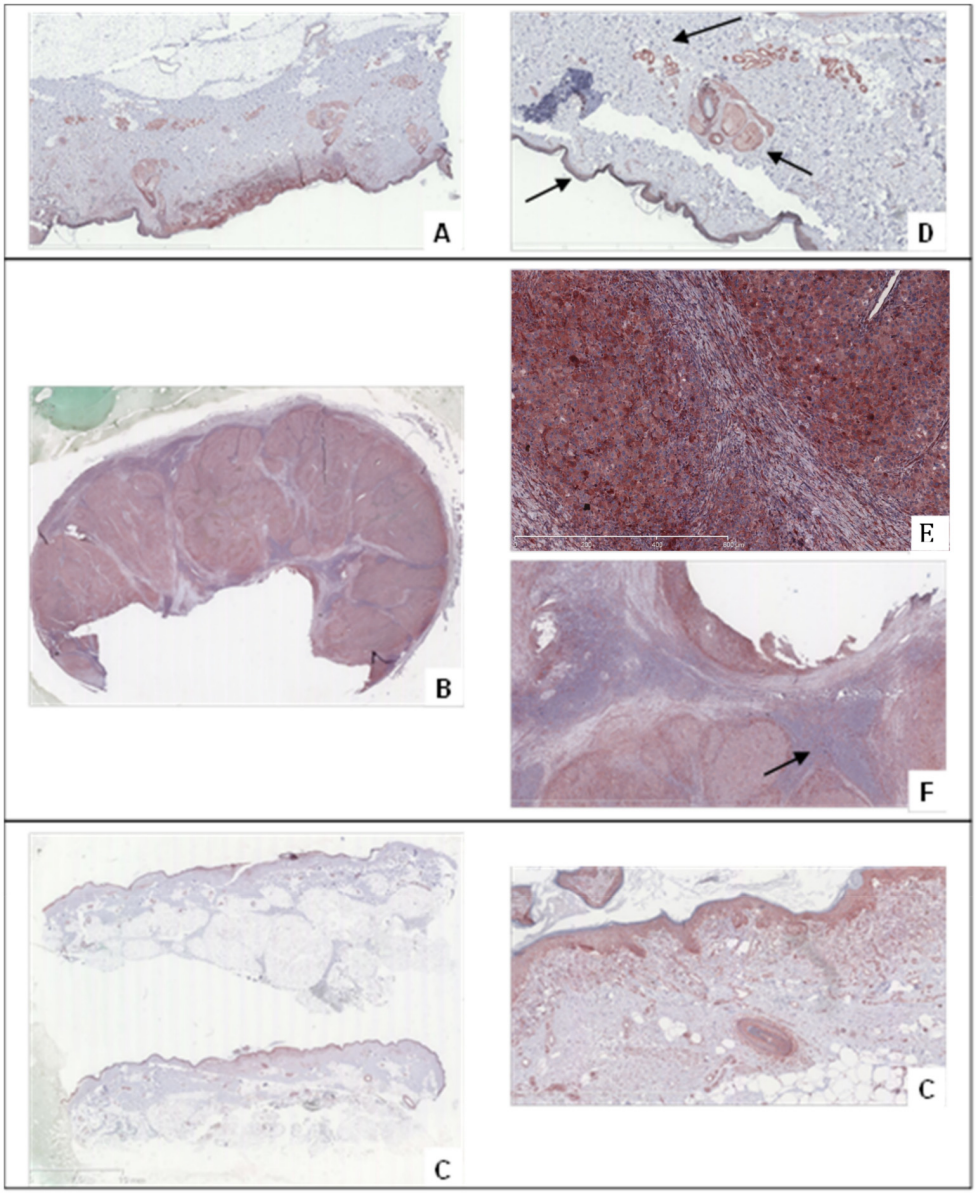
Immunostaining using anti-hMMP-9 murine monoclonal antibody. The antibody ab58803 localizing area of high expression of the integrin in respectively (A) Superficial Spread Melanoma, (B) Metastatic nodes, (C) Lentigo Malignant Melanoma. (D) Background in the immunohistochemical analysis of Superficial Spread Melanoma with staining of epidermis, endothelial cells and sebaceous glands. Immunohistochemical detection of hMMP-9 using ab58803 antibody in a mostly metastatic node, with specific cytoplasmic immunoreactivity in tumor cells (E) and negative results for conjunctive tissue and normal lymphocytes (F).

MMP-9 expression was first investigated in several human tumors from skin by immunohistochemistry using a specific hMMP-9 antibody. Healthy skin was used as MMP-9 negative control. The antibody was able to target active hMMP-9 (83 kDa) and pro-hMMP-9 (92 kDa). Immunohistochemical study revealed that hMMP-9 was overexpressed in melanomas. Strong reactivity was mainly observed in the cytoplasm of numerous tumor cells (Fig 6E). Immunopositivity was also present in the endothelial cells of blood vessels in the tumor environment and disturbing background was also observed in epidermis and in sebaceous glands (Fig 6D). As shown in Fig 6, the hMMP-9 expression, monitored by immunohistochemical analysis, showed an important staining in metastasis nodes whereas no immunoreactivity was detected either in conjunctive tissue or in healthy lymphocytes.

**Fig 7:**
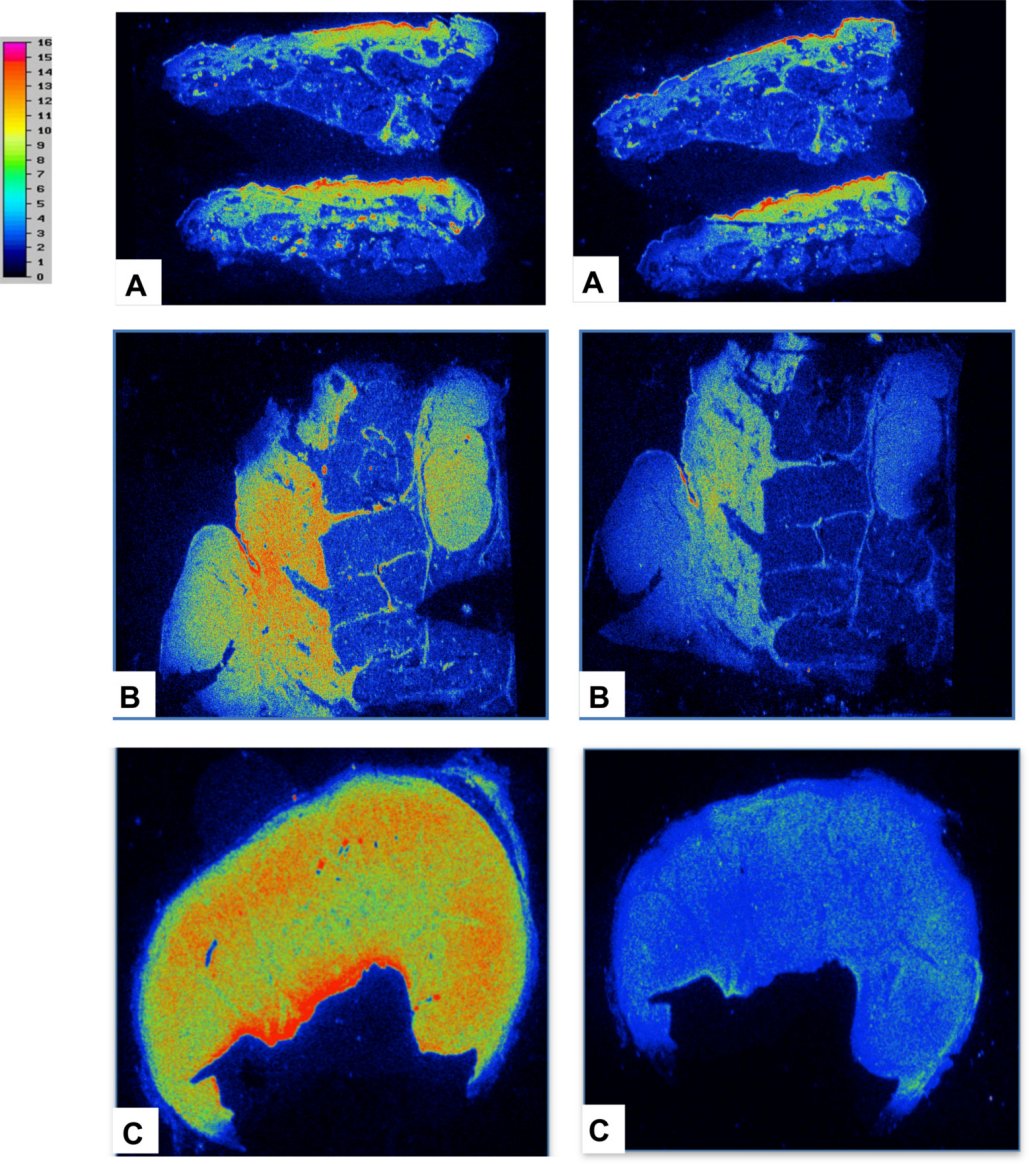
Comparison of the results obtained by radiolabeling of representative tumor tissue sections with ^111^In-DOTA-F3B aptamer (left image) and ^111^In-DOTA-control sequence (right image). The difference of activity seems to increase in a tumor grade-dependent manner. (A) Lentigo malignant melanoma, (B) Nodular melanoma, (C) Mostly metastatic node.

**Fig 8:**
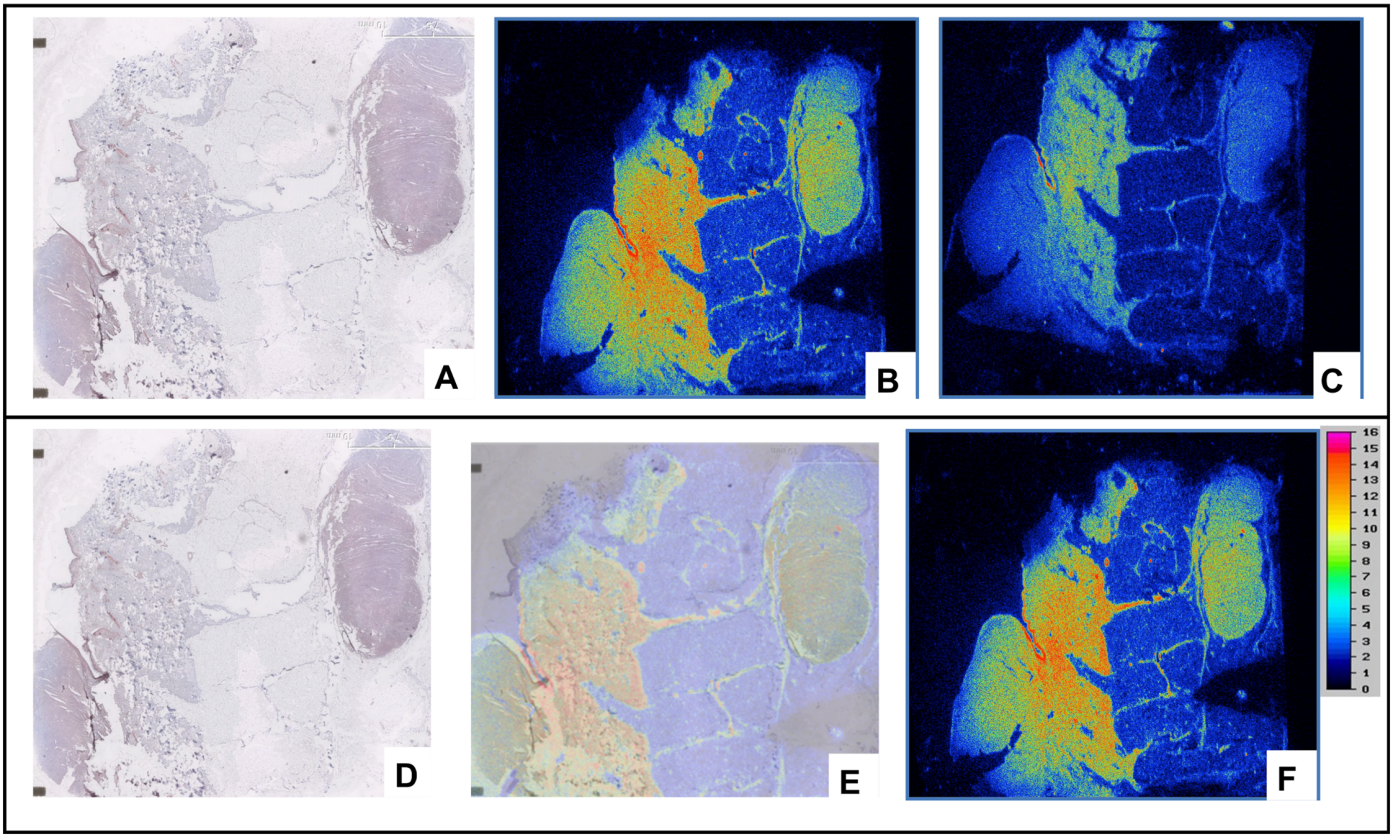
(A) Immunostaining of nodular melanoma using anti-hMMP-9 murine monoclonal antibody ab58803. Radiolabeling of adjacent tissue sections of nodular melanoma with ^111^In-F3B-DOTA (B), and ^111^In-DOTA-control sequence (C). Comparison of the results obtained by radiolabeling with ^111^In-DOTA-F3B and by immunostaining. The results of immunohistochemical images corresponded with area with high activity. (D) Immunostaining of nodular melanoma with ab58803 murin monoclonal antibody anti-human MMP-9. It’s possible to identify the primitive nodular melanoma and an intrahypodermic node (E) Merged Images. (F) Radiolabeling with ^111^In-DOTA-F3B.

For the others primary tumors explored (superficial spread melanoma (pT1b), lentiginous malignant melanoma (pT1a), nodular melanoma (pT4b,N3M1), nodes/metastases), hMMP-9 immunostaining showed a variable but positive expression and the observation was easier in case of infiltrative tumors. The labelling of the adjacent slices of melanoma using ^111^In-DOTA-F3B or control aptamer tended to confirm the results previously obtained in immunostaining. Micro-imager analysis showed a fair agreement with the interaction of the h-MMP-9 monoclonal antibody. The radiolabeled ^111^In-DOTA-F3B aptamer induced a strong signal compare to ^111^In-DOTA-control (Fig 7). One can notice that the difference was weaker for low-grade melanomas. Moreover, in accordance with immunohitochemical analysis, the highest signal was obtained with nodes/metastases. The merger of the obtained images revealed a good colocalization on this sample of nodular melanoma between the binding of the anti-hMMP-9 antibody and the labeling of the tracer (Fig 8). So binding intensities seemed to well correlate with the amount of hMMP-9 detected by immunohistochemistry.

## Discussion

In the field of diagnostic imaging, aptamers have shown great promise as recognition units for tumor targeting [27; 29; 37]. Due to the advantage of their structure and properties (i.e. small size, non-immunogenic, similar binding affinity than antibodies), aptamers binds to their targets with high affinity and specificity. Up to date, limited studies have been performed for *in vivo* tumor targeting using an aptamer labelled with a SPECT radionuclide. In a previous study, our group has demonstrated that radiolabeled F3B aptamer was able to target *in vitro* hMMP-9 protein, a tumor biomarker over-expressed in numerous various malignant tumor. In this study, we go a step further trying to evaluate *ex vivo* and *in vivo* melanoma tumor targeting using fluorescent or isotope labelled specific aptamer directed against hMMP-9 protein.

Due to the instability of small oligonucleotides in biological fluids, a specific aptamer has been truncated down to 36 nucleotides by substitution of RNA residues by 2'-O-methyl derivatives to increase Rnase/nuclease resistance in order to be able to perform *in vivo* studies.

First of all, F3B-DOTA and F3B-Cy5 affinity to hMMP-9 were evaluated using surface plasmon resonance experiments. Functionalized aptamer remained able to bind to hMMP-9 and its pro-form with a strong avidity since competition experiments performed with unmodified aptamer F3B immobilized on the chip in the presence of F3B-DOTA or F3B-Cy5 conjugates with MMP-9 lead to abolish the SPR signal. When optical tagged, the intra-venous injection of Cy5-F3B-aptamer in mice bearing melanoma increased fluorescence signal inside the tumor. This demonstrates an active targeting to hMMP-9 since Cy5-control-sequence did not allow us to localize the tumor. However, since penetration of near infrared light in tissues is limited to a few centimeters [38] with low spatial resolution [39] and limited ability to perform *in vivo* quantitative biodistribution, precise biodistribution of specific aptamer directed against hMMP9 and control aptamer was performed thanks to the immobilization of technetium 99m inside a chelate (MAG3). The use of technetium 99m isotope for radiolabeling aptamer has several advantage including a suitable half-life, appropriate gamma photon energy, availability and low cost. In this study, we efficiently labelled ^99m^Tc-MAG3-aptamer with high radiochemical purity and yield. MAG3 chelator is of particular interest as a bifunctional technetium chelator because it allows forming stable ^99m^TcO core efficiently and rapidly and reduces serum proteins binding [40]. Due to their small size, blood clearance of both aptamers was very fast with a rapid diffusion leading to a maximal tumor uptake of 1.8%ID/g at 1h with a tumor to blood ratio of 9 for ^99m^Tc-MAG-F3B-aptamer. Nevertheless, accumulation and retention in digestive tract at different time points of both ^99m^Tc-MAG3-aptamer were very high (more than 18%ID/g), as frequently observed in previous reports [37; 41]. This important uptake was probably due to the lipophilicity of the radiolabeled compounds [42]. In such context, the detection of melanoma tumor and their metastases would be particularly difficult in the digestive and abdominal region leading us to envisage the use of a more hydrophilic and anionic chelate (i.e. DOTA) which is able to complex a wide variety of imaging or therapeutic radiometals including indium 111 witch could label DOTA at high radiochemical purity in mild condition. In contrast to ^99m^Tc-MAG3-aptamer, the use of DOTA chelator conjugates to the aptamer decrease dramatically the digestive tract uptake (< 2% ID/g at any time points) for ^111^In-DOTA-F3B aptamer and ^111^In-DOTA-control-aptamer. The biodistribution study of ^111^In-DOTA-F3B revealed a slightly tumor uptake at 30 min and 1h post injection compared to the ^99m^Tc-MAG-F3B-aptamer. These results support previous works confirming that various chelators could significantly affect the *in vitro* and *in vivo* biodistribution of the radiolabeled target.

The highest A375 human melanoma tumor uptake of ^111^In-DOTA-F3B aptamer compared to the ^111^In-DOTA-control-sequence was confirmed *ex vivo* by autoradiography and by *scintigraphic images*. Nevertheless, even if human tumor cell lines transplants in athymic mice are widely used for evaluating the targeting efficiency of a potential probe, the murine xenograft model has some limitations for representing the physiopathology and many aspects of human real cancer. In such context, *ex vivo* imaging of human slice of various grade of melanoma tissues have been performed using ^111^In-DOTA-aptamer and compared to hMMP-9 expression by immunostaining. This is an important step to obtain a preliminary indication of the relevant use of indium 111 labelled hMMP-9 aptamer on clinical neoplasms. First of all, our results confirmed the over expression of hMMP-9 in melanoma tumors and were well correlated with the binding intensities of ^111^In-F3B-DOTA even if the anti-hMMP-9 antibody used for immunostaining was raised against the mouse MMP-9 with lower specificity compared to the aptamer F3B. Moreover, hMMP-9 expression and ^111^In-F3B-DOTA binding corroborated to the grade of malignancy whereas the radiolabeled control sequence induced a weaker signal confirming the binding specificity of ^111^In-DOTA-F3B to its target. The presence of DOTA, which is known to be a strong chelator for a variety of beta- or alpha metals such as Lutetium 177, Yttrium 90 or Bismuth 213 allows us to envisage their use in radionuclide therapy. To reach this goal, we are currently investigating the use of a dendritic structure bearing 2 F3Bomf aptamer since our group has recently demonstrated [43] that multivalency on a single dendron structure could highly increase tumor targeting thanks to their cooperative effect [44].
